# IMPROVING MEDICAL STUDENT RECOGNITION AND UNDERSTANDING OF MORAL INJURY IN MILITARY VETERANS

**DOI:** 10.1093/geroni/igac059.1716

**Published:** 2022-12-20

**Authors:** Maribel Rodriguez-Gonzalez, Michael Alan Silverman, Maura Miller, Sandra DiScala, Mythili Bharadwaj, Anthony Beazley

**Affiliations:** Florida Atlantic University, West Palm Beach, Florida, United States; FAU-West Palm Beach VA Medical Center, West Palm Beach, Florida, United States; West Palm Beach VA Medical Center, Jupiter, Florida, United States; West Palm Beach VA Medical Center, West Palm Beach, Florida, United States; West Palm Beach VA Medical Center, West Palm Beach, Florida, United States; West Palm Beach VA Medical Center, West Palm Beach, Florida, United States

## Abstract

Developing a curriculum for medical students is essential in teaching specific skill sets to better care for patients. There are 18,000,000 living military Veterans in America with unique needs not usually included in medical school curriculum. Military personnel are trained to kill or neutralize the enemy, a doctrine that goes against the moral, religious, and societal principle of thou shalt not kill. Combat Veterans are frequently placed in circumstances where they are forced to commit or participate in acts that go against their moral beliefs leading to a dimensional problem called Moral Injury (an affliction of the soul) with accompanying psychological, behavioral, social, and symptoms such as lack of trust, spiritual distress, fatalism, regret, depression, self-loathing, and apathy, etc. and can be a significant problem encountered in older Veterans precipitated by the normative changes of aging. Discussion with more than one hundred second- and third-year medical students over a 3-year period showed that the students were unfamiliar with Moral Injury. The Geriatrics Department at the West Palm Beach VA Medical Center has created an educational module for medical students that focuses on 1) recognizing the symptoms of Mora Injury, 2) how to approach and converse with Veterans including specific questions to ask and what not to say, 3) the importance of a team approach including a chaplain, 4) interventions such as providing forgiveness and life review, and 5) being cognizant of the trust issues of older LGBTQ Veterans. The response of the medical students has been both positive and enthusiastic.

